# Alternative treatment of recurrent *Clostridioides difficile* infection in adults by fecal transplantation: an overview of phase I–IV studies from Clinicaltrials.gov

**DOI:** 10.3389/fmicb.2024.1374774

**Published:** 2024-05-09

**Authors:** Najla A. Obaid

**Affiliations:** Pharmaceutical Sciences Department, College of Pharmacy, Umm Al-Qura University, Makkah, Saudi Arabia

**Keywords:** *Clostridioides difficile* infection, fecal microbiota transplant, full-spectrum microbiota, clinical trials, recurrent CDI (rCDI)

## Abstract

**Background:**

Fecal microbiota transplantation (FMT) is an interventional approach to treat chronic and recurrent *Clostridioides difficile* infection (CDI). However, there is insufficient evidence regarding its effectiveness and safety. Clinical trials have been conducted to inspect the safety and effectiveness of FMT with and without comparison to pharmacological treatments.

**Aim:**

This review explored the treatment of CDI in adults using FMT and evaluated the safety of this intervention based on phase I–IV studies registered on Clinicaltrials.gov.

**Method:**

A comprehensive search of Clinicaltrials.gov was conducted to identify relevant studies that investigated CDI in adults. Data on study type, study design, sample size, intervention details, and outcomes related to FMT were examined and evaluated.

**Results:**

In total, 13 clinical trials on FMT for CDI published through 17 November 2023 were identified, all of which were interventional studies. The investigation focused on both terminated and completed studies. Basic and advanced outcome measures were examined.

**Conclusion:**

Some studies were terminated during phase II, and FMT was less effective than antibiotics such as vancomycin and fidaxomicin. However, colonoscopy and oral FMT were explored in several completed studies with promising results, but the evidence remains limited and inconclusive.

## 1 Introduction

*Clostridioides difficile* infection (CDI) is a concerning global health issue, and the incidence of hospital-acquired CDI has substantially increased in recent years globally (Wiegand et al., 2012). CDI remains a major contributor to diarrhea in healthcare settings, even with established prevention and treatment protocols (Hensgens et al., 2014). CDI places a major economic burden on the healthcare system due to prolonged hospitalization and on patients due to comorbidities, repeated infections, extended length of stay, increased treatment costs, and indirect societal costs (Gupta and Ananthakrishnan, 2021). In total, 30% of patients with CDI develop recurrence and are associated with 33% higher hazards of death (Olsen et al., 2015). The difficulty of CDI treatment illustrates the need to prevent recurrence and develop effective therapies.

Updated recommendations on CDI treatment and prevention have been issued by the Infectious Diseases Society of America and Society for Healthcare Epidemiology of America (IDSA), the American College of Gastroenterology, and the European Society of Clinical Microbiology and Infectious Diseases (Bainum et al., 2023). CDI treatment has progressed, with vancomycin and fidaxomicin being the primary treatments, whereas metronidazole is only recommended for non-severe cases when patients are unable to obtain or to be treated with oral vancomycin or fidaxomicin (Jarmo et al., 2020). Some studies concluded that fidaxomicin has advantages over vancomycin in terms of reducing recurrence and that fidaxomicin is cost-effective as a first-line therapy (Louie et al., 2011; Cornely et al., 2012; Beinortas et al., 2018; Mikamo et al., 2018). The University of Pittsburgh Medical Center released guidelines (2018) to use fidaxomicin for the first recurrence of CDI or subsequent recurrences (Bariola, 2019). However, fecal microbiota transplantation (FMT) has been recommended by many studies for treating CDI recurrence (Bariola, 2019; Jarmo et al., 2020). Recently, some FMT products were approved by the Food and Drug Administration (FDA) for treating recurrent CDI (Aschenbrenner, 2023; Jain et al., 2023). Strict clinical studies, good manufacturing processes, and donor and pathogen screening are important ways that this approach differs from live biotherapeutic products (LBPs) and traditional FMT (Monday et al., 2024). Donor banks are not established to guarantee that the donors' health is in good condition. To prevent CDI recurrence, the FDA approved a live fecal microbiota product called “Rebyota” (Aschenbrenner, 2023). This product was specifically approved after the failure of antibiotic treatment and the recurrence of CDI in adults. This product provides a potential solution to recurrent CDI following the failure of antibiotic treatment (Cornely et al., 2012). A novel FMT product named Vowst was also approved by the FDA as a prophylactic therapy to prevent the recurrence of CDI. Vowst consists of live fecal microbiota spores and works by re-establishing the gut microbiota and providing a better microbiome for the patient (Jain et al., 2023). Although an endoscopic technique is the recommended mode of delivery for FMT, limited research has examined the potential of frozen oral tablets for FMT. FMT formulated as frozen tablets or capsules is not inferior to FMT performed via colonoscopy (Jain et al., 2023). The approval of Vowst represented a major advancement in the prevention of recurrent CDI (Jain et al., 2023). There are uncertainties regarding the effectiveness of this product for CDI patients other than the participants in its clinical trials, and a prior study recommended that the donor screening process should be improved (Jain et al., 2023).

Approved FMT products are the recommended options for recurrent CDI. Current treatment options for CDI include vancomycin and fidaxomicin as the primary treatments (Jarmo et al., 2020). Metronidazole is reserved for mild cases, and bezlotoxumab, a monoclonal antibody against *C. difficile* toxin B, is used as an additional treatment for recurrent CDI (Jarmo et al., 2020). Fidaxomicin and FMT are more expensive but more effective in preventing recurrences, and thus, they are becoming the standard of care for CDI (Gupta and Ananthakrishnan, 2021). However, there are limited data on the preferred administration route of FMT, the cost-effectiveness of preventing recurrent FMT, and the use of unconventional therapies such as bezlotoxumab. This review explored the latest update on the prevention of CDI recurrence and the effectiveness of FMT treatment by analyzing the Clinicaltrials.gov dataset.

## 2 Methods

### 2.1 Search strategy

On 17 November 2023, a search was conducted on ClinicalTrials.gov to identify relevant studies using the single search term “*Clostridioides difficile* infection recurrence” for the disease or condition together with “fecal microbiota transplant.”

### 2.2 Search results for the review

**Figure 1:**
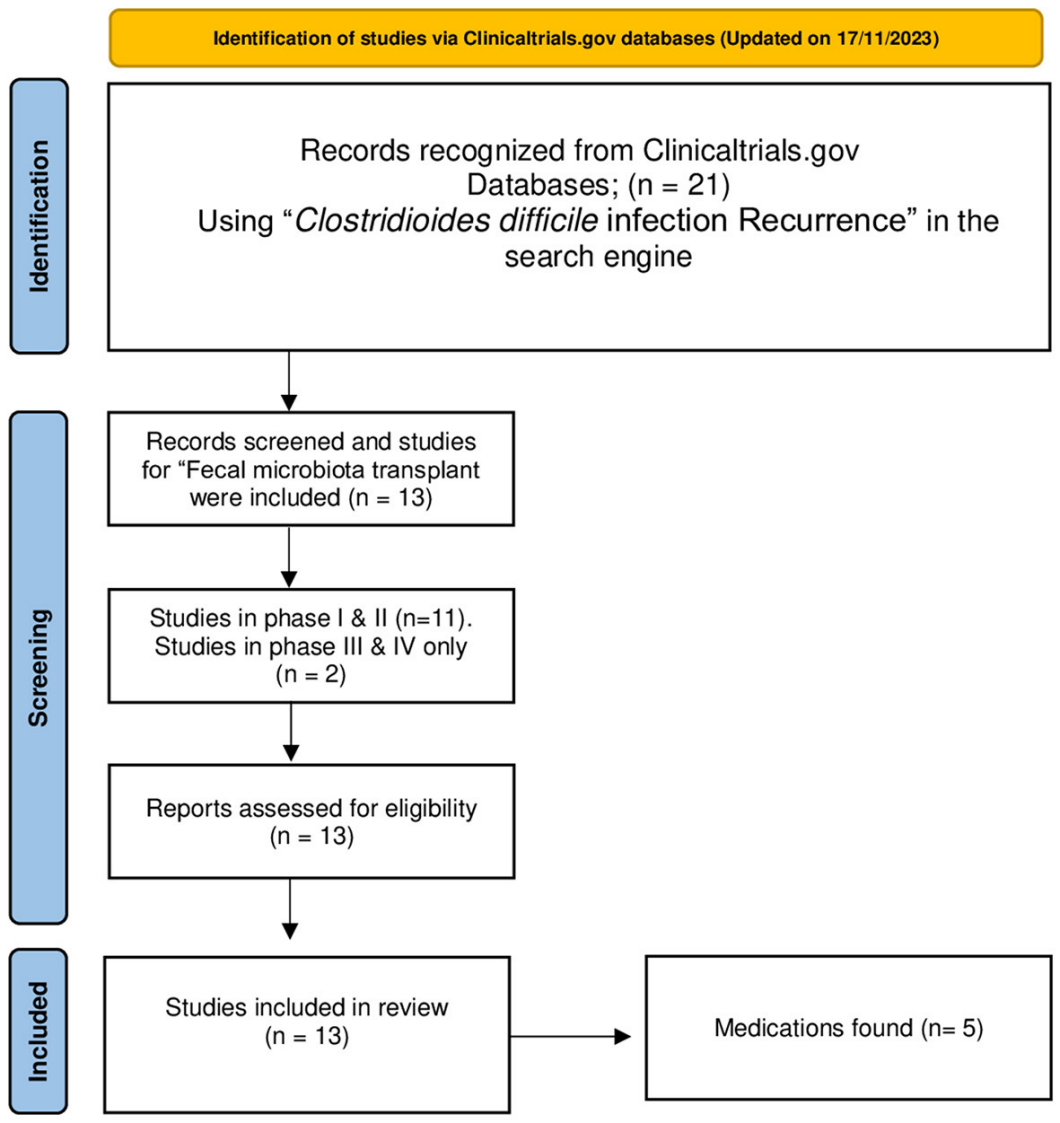
PRISMA flow chart of the identification, screening, and inclusion of studies in this review. Adopted from Page et al. (2021).

Clinical trials of any phase that used FMT as an observational measure were eligible for inclusion, and other trials were excluded (Figure 1).

### 2.3 Extracted data from the screened database

All study information, such as study title, study status, study type, intervention details, characteristics of the studies, and outcomes, was extracted from the database.

## 3 Results

### 3.1 The number of studies returned by the search

In total, 21 clinical trials were identified in the search. Of these, only 13 clinical trials of any phase that performed “fecal microbiota transplant” in adults for recurrent CDI were included in this review.

### **3.2 The characteristics** of the clinical trials

These 13 studies mainly assessed recurrent CDI in patients aged 18 years and older, including elderly patients. Only six trials in the registry were completed studies with results. These completed studies included 1,164 participants in total. The remaining trials were terminated for several reasons, such as administrative reasons, a lack of available funds for follow-up, ineffectiveness, and the investigators no longer considering FMT products for CDI. Completed details are presented in Table 1.

**Table 1:** The characteristics of the clinical trials (from ClinicalTrials.gov 17th November 2023).

	**Title**	**Status**	**Conditions**	**Intervention**	**Phase**	***n***
1	Penn Microbiome Therapy for Recurrent Clostridium Difficile Infection	Terminated	Recurrent *Clostridium difficile* Infection	• Penn Microbiome Therapy – 001 • Penn Microbiome Therapy – 002 • Penn Microbiome Therapy - 003	2	9
2	FMT Versus Antimicrobials for Initial Treatment of Recurrent CDI	Terminated	*Clostridium difficile* Infection	Biological FMT and antimicrobial	2	6
3	A Prospective Trial of Frozen-and-Thawed Fecal Microbiota Transplantation for Recurrent Clostridium Difficile Infection	Terminated	*Clostridium difficile*	Frozen fecal microbiota	2	140
4	Dose Ranging Study of the Safety and Efficacy of Orally Administered Lyophilized Fecal Microbiota Product (PRIM-DJ2727) for the Treatment of Recurrent Clostridium Difficile Infection (CDI)	Terminated	Recurrent C. *difficile* Infection	Low (collected from 50-g of stool for 2 consecutive days), mild (collected from 100-g of stool on the 1st day and then from 50 g of stool on the consecutive day) and high (collected from 100-g of stool for 2 consecutive days) fecal microbiota dose	2	17
5	Safety of FMT: OpenBiome Outcomes and Longitudinal Follow-up (STOOL) for Recurrent Clostridium Difficile Infection	Terminated	• *Clostridium difficile*	Fecal microbiota preparation (frozen processed fecal material)	2	17
6	Microbiota Restoration Therapy for Recurrent Clostridium Difficile Infection (PUNCHCD2)	Completed	• Enterocolitis *Clostridium difficile* Recurrent	• RBX2660 (microbiota suspension)	2	150
7	Efficacy, Safety, and Tolerability Study of Oral Full-Spectrum MicrobiotaTM (CP101) in Subjects With Recurrent C. Diff (PRISM3)	Completed	• *Clostridium difficile* Infection recurrent	• Full-spectrum microbiota capsule	2	206
8	Microbiota Restoration Therapy for Recurrent *Clostridium difficile* Infection	Completed	• *Clostridium difficile* Infection	• Rebiotix RBX2660 (microbiota suspension)	2	272
9	The ICON Study: Outcomes After FMT for Patients With IBD and CDI	Completed	Inflammatory bowel diseases, *Clostridium difficile* Infection	• Fecal microbiota transplantation	1 and 2	50
10	Open-Label Extension of CP101 Trials Evaluating Oral Full-Spectrum Microbiota™ (CP101) in Subjects With Recurrence of Clostridium Difficile Infection (PRISM-EXT)	Completed	*Clostridium difficile* Infection Recurrent *Clostridium difficile* Infection	Full-spectrum microbiota	2	132
11	Microbiota Restoration Therapy for Recurrent Clostridium Difficile-associated Diarrhea (PUNCH CD)	Completed	Recurrent *Clostridium difficile* Infection	RBX2660 (microbiota suspension)	2	34
12	A Trial of CP101 for the Prevention of Recurrent CDI (PRISM4) (PRISM4)	Terminated	Recurrent *Clostridium difficile* Infection	CP101 (an investigational microbiome therapeutic designed to deliver a complete and functional microbiome)	3	19
13	Microbiota Restoration Therapy for Recurrent Clostridium Difficile Infection (PUNCHCD3)	Completed	*Clostridium difficile* Infection (CDI)	RBX2660 microbiota suspension	3	320

n, number of participants.

### 3.3 Outcome measures

Most of the clinical trials focused on the clinical resolution of symptoms. The primary outcomes included diarrhea, abdominal pain, length of hospital stay (90 days), mortality within 90 days, ≤ 4 stools per day for 2 days with no stool categorized as type 7 on the Bristol Stool Scale, no positive result on enzyme immunoassay for *C. difficile* toxin after treatment, no recurrence of the infection within 8 weeks after the transplant, and no additional antibiotic prescription. The secondary outcomes included the evaluation of the safety of FMT and the absence of adverse effects; measurement of serious adverse events (SAEs), including death or life-threatening events; prolonged hospitalization; and significant incapacity of normal life function. Only two studies included a placebo group for comparison, and one study compared FMT to antibiotic treatment. A complete list is provided in Table 2.

**Table 2:** Outcome measures with the prevention of recurrent CDI with FMT (from ClinicalTrials.gov 17th November 2023).

**Product used**	**Outcome measures**	**The presence of the placebo group**
FMT G3 capsules	- A comparison between the stool microbiome with and without FMT administration - The number of FMT pills administered after the completion of a course of oral vancomycin. - The emergence of adverse events - The incidence of gastrointestinal symptoms - CDI recurrence within 60 days - The number of hospital readmissions	
50 g of fecal material suspended in bacteriostatic normal saline and glycerol	−90 days of clinical resolution of symptoms (diarrhea and abdominal pain) - Patients' hospital length of stay after 90 days of transplantation. - Re-admission of patients to the hospital for recurrent CDI - Mortality rate	
1 enema RBX2660 (microbiota suspension) A suspension of intestinal microbes	- The absence of the syptoms at 56 days after FMT - Successful treatment between the groups (I enema of RBX2660 and 1 enema of placebo) - Validated SF-36 scale was used by the study to identify the changes to quality of life. - Time to CDI recurrence between groups using Kaplan–Meier analysis	Yes
Frozen fecal microbiota kept at−20°C and thawed prior treatment	- No CDI recurrence within 13 weeks - The evaluation of the safety of FMT for any serious adverse event up to 13 weeks	
Single and two doses of Penn Microbiome Therapy (PMT) PMT-001 PMT-002 PMT-003	- The number of patients with clinical resolution of diarrhea within 8 weeks - Colectomy within 30 days after FMT - Hospital length stay within 30 days of FMT - The number of readmissions to hospital within 60 days - Mortality rate (following 30 days then 90 days of FMT)	
Frozen processed human fecal material	- Measuring for absence or presence of serious adverse events post 6 weeks of FMT - Measuring for absence or presence of serious adverse events post 6 weeks to 1 year of FMT	
Low, mid, and high fecal microbiota dose	- Safety assessed by the number of participants with nausea, vomiting, diarrhea, bloating, and constipation post FMT - The number of participants with recurrent CDI	
CP101 Full spectrum microbiota capsule	- The number of participants with absence of recurrence more than 8 weeks - The number of participants with occurrence of treatment adverse event	Yes
Fecal microbiota transplantation	- Recurrence of CDI within 8 weeks - Positive stool testing for *C. difficile* via polymerase chain reaction	

### 3.4 Success rate and the safety of FMT

FMT was performed using different formulations, as presented in Table 3, together with a description of the success rate and safety (mortality and SAEs). The success rate varied between the primary outcomes measured in these studies. Some studies focused on the absence of CDI recurrence within 8 or 24 weeks. Other studies examined the occurrence of symptoms such as nausea, vomiting, and constipation. Many studies recorded a mortality rate of 0%. All studies reported SAEs, excluding the one that did not measure SAEs.

**Table 3:** Successful rate and indication for safety (from ClinicalTrials.gov 17th November 2023).

**Product used**	**Percentage of successful for FMT**	**Indications for safety**
1 enema RBX2660 (microbiota suspension)	55.6% with overall efficacy (88.8%) (Dubberke et al., 2018)	Mortality 16.67%
		SAE 42.86%
50 g of the fecal material suspended in bacteriostatic normal saline and glycerol	50%	Mortality 0%
Frozen fecal microbiota kept at −20°C and thawed prior to treatment	89.47%	Mortality 8.3%
		SAE 12.03%
Single and two doses of Penn Microbiome Therapy (PMT) PMT-001 PMT-002 PMT-003	20% for a single PMT 50% for two PMT	Mortality 0% SAE for single PMT 60% SAE for two PMT 75%
Fecal microbiota preparation (frozen processed fecal material)	Not specified	Mortality 0%
		SAE 26.67%
Low, mid, and high fecal microbiota dose	Safety assessed by the percentage of participants with nausea (66.7% mid FMT and 12.5% high FMT), vomiting (33.3% mid FMT), diarrhea (25% low FMT and 25% high FMT), bloating (25% high FMT), and constipation (12.5% FMT).	Mortality 0%
	Recurrent CDI (50% low FMT and 25% high FMT)	SAE low FMT 25%
CP101 Full-spectrum microbiota capsule	The absence of recurrent CDI in week 8: 74.5%	Mortality 0.96%
	The absence of recurrent CDI in week 24: 73.5%	SAE 15.38%
Full spectrum microbiota	The absence of recurrent CDI in week 8: 80%	Mortality 0.76%
	The absence of recurrent CDI in week 24: 78.8%	SAE 12.88%
Fecal microbiota transplantation	FMT failure within 8 weeks 8.2%	Mortality 0%
	Colonized with *C.difficile* 10.2%	SAE 6%

SAE, serious adverse event.

## 4 Discussion

Disruption of the intestinal microbiome contributes to many human conditions and symptoms (Weiss and Hennet, 2017). Recurrence of CDI is a serious condition caused by disruption of the intestinal microbiome because of the use of antibiotics (Seekatz et al., 2022). Hospitalized patients who have been prescribed antibiotics for a long period have a high risk of *C. difficile* infection, and *C. difficile* toxins cause abdominal pain and diarrhea (Goldberg et al., 2015). Antibiotics prescribed as standard treatments for both acute and recurring illnesses do not treat abdominal syndromes such as dysbiosis; in fact, they frequently make them worse (Seekatz et al., 2022). Monoclonal antibodies, such as bezlotoxumab, can reduce the risk of recurrences, but they do not treat the underlying dysbiosis (Wilcox et al., 2017; Seekatz et al., 2022). They are recommended to be used in conjunction with standard-of-care antibiotics for preventing recurrent CDI in patients at high risk of recurrence. FMT is a developing therapeutic approach in which the gut microbiome of a recipient is restored by introducing bacteria from a healthy donor's stool into the recipient's gut. There are several FMT preparation methods and routes of administration. The effectiveness of FMT is considered to vary between different routes (Gough et al., 2011). The oldest technique followed for FMT is the direct infusion of donor stool through colonoscopy, and it is considered to be simple, safe, and 92% effective in the treatment of recurrent CDI (Kelly et al., 2012). Other FMT administration methods include nasogastric or nasoenteric tube and enema (Brandt and Aroniadis, 2013). Several barriers to the use of FMT have emerged, such as determining the features of a healthy microbiome, assuring the receiver's safety in terms of long-term consequences, sufficiently monitoring the recipient of the fecal material, and attaining high-quality control (Kim and Gluck, 2019). Therefore, the preferred administration method and the route of FMT depend on overcoming these barriers to ensure treatment efficacy.

FMT delivered via intestinal suspensions is reported to provide a high rate of symptom resolution, reaching 92% in some studies (Gough et al., 2011). Most of the clinical trials included in this review were performed using fecal suspensions delivered via enema and colonoscopy. RBX2660 is one of the FMT preparations used in the clinical trials described in this review (Table 2), and it was approved by the FDA under the name Rebyota (Kim et al., 2023). The potential of this preparation to restore the microbiome can alleviate CDI recurrence, and it was more effective than the placebo in terms of restoring the microbiome (Blount et al., 2019). The standardization of the material for FMT preparation from the donor can significantly simplify the clinical use of FMT for recurrent CDI (Hamilton et al., 2012). The transplant material is prepared from a donor stool from two sources: current patients and universal donors from a stool bank (Edelstein et al., 2016). The major drawback in the use of FMT for recurrent CDI is the lack of standardization regarding the preparation process and administration techniques (Berry and Khanna, 2023). In addition, patients who have completed antibiotic therapy for recurrent CDI and who are 18 years and older can receive Rebyota as a prophylactic biotherapeutic treatment (Berry and Khanna, 2023). It should be taken rectally and needs to be administered only once (Kim et al., 2023).

FMT can also be performed using prepared capsules. FMT capsule formulations combine the ease of administration of an antibiotic with the efficacy of FMT for treating recurrent CDI (Varga et al., 2021). One study in this review (Table 2) examined FMT performed using prepared capsules, and two studies delivered a full-spectrum microbiota using an oral formulation (CP101). One study was terminated in phase III, and the primary outcomes were not analyzed because no patients completed treatment (Table 1). Another study using oral CP101 was completed, with the primary outcome being achieved in 80.3% of patients (Table 3). The third study using CP101 presented some results assessing its efficacy (primary outcome) and safety (secondary outcome) in subjects with recurrent CDI (Table 2). Based on the percentage of participants with adverse events after oral FMT, safety was not achieved in comparison with the placebo, and many adverse events from recurrent CDI were observed. The Penn microbiome therapy for recurrent CDI was examined in a terminated clinical trial that administered three preparations (the Penn microbiome therapy 001 [enema product], the Penn microbiome therapy 002 [suspension product], and the Penn microbiome therapy 003 [capsule product]), as detailed in Tables 1, 2. This study observed a low rate of clinical resolution with one or two doses. However, the mortality rate was 0% (Table 3). From these results, the achievement of the primary and secondary outcomes was inconsistent for oral FMT preparations, and further clinical trials are needed.

Regarding the cost-effectiveness of preventing CDI recurrence, FMT performed via colonoscopy was identified as the most cost-effective approach in one clinical trial included in this review (Konijeti et al., 2014). The study recorded cure rates exceeding 88.4% and CDI recurrence rates lower than 14.9% with colonoscopy-based FMT compared with antibiotics (Konijeti et al., 2014). This finding was also suggested in a study published nearly 10 years before this review. The recently approved orally administered FMT named Vowst was examined in a clinical trial (SER-109) and then received FDA approval on 26 April 2023 (Jain et al., 2023). This product is non-invasive and more patient-friendly, and it minimizes the risk of iatrogenic consequences (Jain et al., 2023). Regardless of the number of past CDIs by age or antibiotic type (vancomycin or fidaxomicin), a remarkable clinical response was observed at week 8 in 91.3% of patients receiving Vowst, and the rate increased to 94.6% at week 24 (Khanna et al., 2022). Moreover, 87.6% of patients in the Vowst group were free from recurring CDI at the end of 8 weeks in comparison to 60.2% of participants in the placebo group, indicating that Vowst decreased CDI recurrence. Along with having a better safety profile, Vowst also caused comparatively mild-to-moderate transient side effects. The rigorous inclusion criteria used in the clinical trials (phase 1) raise some uncertainties about the specific efficacy of Vowst in patients with CDI outside those who participated in the trials (McGovern et al., 2021). For instance, patients with cancer, those requiring additional antibacterial medication (surgical prophylaxis and urinary tract infections), and those with a history of inflammatory bowel disease were excluded, as were female participants who were pregnant, nursing, or lactating (Jain et al., 2023). Regarding the safety profile, mild-to-moderate adverse effects were noted in a phase III trial (McGovern et al., 2021). We can conclude that the better safety profile along with a low number of FMT courses (either coloscopy or oral) can be considered more cost-effective than expensive antibiotics. However, economic analyses need to be conducted to explore the benefit and effectiveness of oral capsule formulations for FMT (Jain et al., 2023), as the cost-effectiveness of Rebyota and Vowst was not examined in their clinical trials.

This review had several limitations. Because the variables linked to the FMT technique were inconsistently classified across trials, operational definitions were established beforehand to facilitate data abstraction. Limited clinical trials progressed to completion, and from these publications, data on the techniques of FMT and cost-effectiveness were not sufficiently recorded. In addition, three trials were terminated without generating data because no patients could be analyzed.

## 5 Conclusion

Based on the records of FMT treatment for recurrent CDI from ClinicalTrials.gov, the completed clinical trials recorded high clinical resolution rates of CDI symptoms with mild-to-moderate SAEs but extremely low mortality rates. However, resolution rates can be affected by variations in the FMT process. Colonoscopy in the earliest studies achieved a high success rate, and even higher success rates were achieved in later trials using oral formulations for full-spectrum FMT. Furthermore, the data indicate that, in cases in which conventional therapies have failed, FMT using FDA-approved products could be an extremely safe and effective treatment for recurrent CDI. These methods proved to be differentially effective, suggesting that personalized approaches to FMT may enhance its success rate. Moreover, several studies highlighted the potential cost-effectiveness of FMT, which is a significant consideration given the economic burden of CDI. The analysis encompassed a diverse range of interventional studies, which demonstrated a notable variance in success rates, reflecting the heterogeneity of FMT applications and patient demographics. Despite these variations, the collective data highlight a trend toward positive outcomes with the use of FMT in the management of CDI.

In conclusion, the analyzed database substantiates that FMT is a safe and effective treatment for recurrent CDI. It highlights the potential for FMT to be incorporated more prominently into clinical practice as a therapeutic strategy against CDI. Future research should focus on standardizing FMT procedures, optimizing delivery methods, and monitoring for long term to fully exploit its therapeutic benefits and ensure the consistent safety and effectiveness of FMT.

## Data availability statement

The raw data supporting the conclusions of this article will be made available by the authors, without undue reservation.

## Author contributions

NO: Conceptualization, Data curation, Formal analysis, Investigation, Methodology, Writing—original draft, Writing—review & editing.
